# Diagnostic Utility of Extended Focused Assessment With Sonography in Trauma (eFAST) in Hemodynamically Stable Patients With Blunt Thoracoabdominal Injury: A Prospective Study

**DOI:** 10.7759/cureus.106015

**Published:** 2026-03-28

**Authors:** Utkarsh Bhandari, Sanjay Kumar Jain, Ashish Tiwari, Deepak Nayak, Pratyush Kaithwas, Utsav Gupta

**Affiliations:** 1 General Surgery, Gandhi Medical College, Bhopal, IND

**Keywords:** abdominal blunt trauma, blunt trauma chest, emergency medicine and trauma, point-of-care ultrasound (pocus), trauma management

## Abstract

Background

Extended focused assessment with sonography in trauma (eFAST) has become an important component of trauma evaluation for rapid bedside detection of thoracic and intra-abdominal injuries. Although its role in hemodynamically unstable trauma is well established, prospective evidence evaluating its diagnostic value in hemodynamically stable patients with blunt thoracoabdominal trauma remains limited. This study aimed to assess the diagnostic utility of eFAST in stable blunt trauma patients by examining its concordance with final hospital discharge diagnosis and comparing its detection performance with chest radiography for thoracic injuries.

Methods

A prospective observational study was conducted on 120 hemodynamically stable patients presenting with blunt thoracic and/or abdominal trauma between August 2022 and March 2024. All patients underwent standardized clinical assessment, chest radiography, and bedside eFAST examination. Final hospital discharge diagnosis, incorporating operative findings, intercostal chest tube findings, and clinical course with follow-up imaging when indicated, served as the composite reference standard. Diagnostic performance of ultrasound for thoracic and abdominal injuries was evaluated, and detection rates were compared with chest radiography.

Results

Among the 120 patients included in the study, 78 had thoracic injuries, and 48 had intra-abdominal injuries based on final hospital diagnosis. eFAST detected 68 of 78 thoracic injuries, yielding a sensitivity of 87.2% (95% CI 77.9-93.4) and specificity of 100% (95% CI 91.6-100). For abdominal injuries, ultrasound detected 46 of 48 cases, corresponding to a sensitivity of 95.8% (95% CI 85.7-99.5) and specificity of 100% (95% CI 94.9-100). Chest radiography identified 32 of 78 thoracic injuries (41.0%). The difference in detection between ultrasound and chest radiography was statistically significant (p < 0.001). Ultrasound findings also demonstrated a strong correlation with subsequent management decisions, as all patients requiring intercostal chest tube insertion or exploratory laparotomy had positive ultrasound findings.

Conclusions

eFAST is a reliable and effective bedside imaging modality for evaluating hemodynamically stable patients with blunt thoracoabdominal trauma. It demonstrates high diagnostic accuracy for both thoracic and abdominal injuries and significantly outperforms chest radiography in detecting thoracic trauma. Its strong correlation with management decisions further supports the role of eFAST as a valuable triage adjunct in trauma care, particularly in resource-limited settings.

## Introduction

Trauma remains a leading cause of morbidity and mortality worldwide, accounting for nearly 4.4 million deaths annually, particularly among young adults [1,2]. Blunt thoracoabdominal injuries account for a substantial proportion of trauma admissions and may result in life-threatening complications such as pneumothorax, hemothorax, hemoperitoneum, and hollow viscus injury [3]. Early identification of these conditions is essential to guide timely intervention and optimize outcomes.

Extended focused assessment with sonography in trauma has become an integral component of modern trauma evaluation [4,5]. By enabling rapid bedside assessment for pleural, pericardial, and peritoneal free fluid or air, it provides real-time information that supports early triage and resuscitation decisions [6,7]. Its portability, repeatability, and lack of ionizing radiation make it particularly attractive in emergency settings.

While the role of ultrasound in hemodynamically unstable trauma patients is well established [6,8], its utility in stable blunt thoracoabdominal injury remains less clearly defined. Computed tomography is often considered the preferred imaging modality in stable patients because of its ability to characterize organ injury in detail [9]. However, CT availability may be limited in certain institutions, particularly in resource-constrained environments where patient volume and logistical factors influence imaging pathways [2,10].

Chest radiography and clinical examination remain standard components of initial thoracic trauma evaluation. However, supine radiographs may fail to detect small pneumothoraces and hemothoraces [11]. In contrast, lung ultrasound has demonstrated superior sensitivity for pleural pathology in trauma settings [12,13].

This study aimed to evaluate the clinical utility of extended focused assessment with sonography in trauma in hemodynamically stable patients with blunt thoracoabdominal injury. Specifically, we assessed its concordance with final hospital discharge diagnosis, compared its detection performance with chest radiography, and examined its association with subsequent management decisions.

## Materials and methods

A prospective observational single-center study was conducted at Gandhi Medical College and Hamidia Hospital, Bhopal, Madhya Pradesh, India, between August 2022 and March 2024. A total of 120 hemodynamically stable patients presenting with blunt thoracic and/or abdominal trauma were enrolled. The sample size was calculated based on the expected sensitivity of extended focused assessment with sonography in trauma for detecting thoracoabdominal injuries reported in previous studies. Assuming an expected sensitivity of approximately 85% for eFAST in detecting clinically significant thoracoabdominal injuries [6,12], with a confidence level of 95% and a margin of error of 7%, the minimum required sample size was estimated to be approximately 110 patients using standard formulas for estimation of a single proportion in diagnostic studies. To account for possible exclusions and incomplete data, a total of 120 patients were enrolled in the study.

Hemodynamic stability was defined as systolic blood pressure greater than 100 mmHg, pulse rate less than 110 beats per minute, oxygen saturation greater than 94 percent on room air, and Glasgow Coma Scale score greater than 13. Patients with penetrating thoracic or abdominal injuries, those requiring immediate surgical exploration upon arrival, or those with extensive subcutaneous emphysema limiting ultrasound evaluation were excluded from the study.

All patients underwent standardized trauma assessment according to Advanced Trauma Life Support principles. A detailed clinical examination of the thorax and abdomen was performed, and findings were documented prior to imaging. Each patient underwent supine anteroposterior chest radiography followed by bedside extended focused assessment with sonography in trauma. Ultrasound examinations were performed using a portable ultrasound machine available in the emergency department, utilizing a curvilinear probe (2-5 MHz) for abdominal assessment and a high-frequency linear probe (7-12 MHz) for thoracic evaluation. Examinations were performed by trained clinicians, including surgical residents and radiology personnel with prior experience in FAST/eFAST, who had undergone supervised training in trauma ultrasound protocols. The examination included evaluation of bilateral pleural spaces for pneumothorax and hemothorax, assessment of the pericardial window, and assessment of intraperitoneal spaces including the hepatorenal (Morison’s pouch), splenorenal, pelvic (pouch of Douglas), and perisplenic regions for free fluid.

Ultrasound findings were categorized as thoracic positive or negative and abdominal positive or negative based on the presence of pleural air, pleural fluid, pericardial effusion, or intraperitoneal free fluid. All examinations were interpreted in real time at the bedside by the performing clinician, and no independent blinded review was performed. Final hospital discharge diagnosis was considered the composite reference standard. This included operative findings in patients undergoing exploratory laparotomy, findings during intercostal chest tube insertion, and documented clinical progression with follow-up imaging when indicated during hospitalization. The use of a composite reference standard may introduce verification bias, which has been acknowledged. Computed tomography was not routinely performed in this cohort as per institutional protocol for stable trauma patients.

The primary outcome was concordance of ultrasound findings with final hospital discharge diagnosis for detection of clinically significant thoracic and intra-abdominal injuries, defined as injuries requiring therapeutic intervention (such as intercostal chest tube insertion or exploratory laparotomy) or close clinical monitoring during hospitalization. Secondary outcomes included comparison of detection rates between ultrasound and chest radiography for thoracic injuries and correlation of ultrasound findings with subsequent management decisions.

Categorical variables were expressed as frequencies and percentages. Detection rates between ultrasound and chest radiography were compared using McNemar’s test. A p-value less than 0.05 was considered statistically significant. Statistical analysis was performed using IBM SPSS Statistics for Windows, Version 26.0 (IBM Corp., Armonk, NY, USA).

This study was approved by the Institutional Ethics Committee of Gandhi Medical College and Associated Hospitals, Bhopal (IEC Letter No. 32314/MC/IEC/2022; reviewed August 18, 2022). Written informed consent was obtained from all participants.

## Results

A total of 120 hemodynamically stable patients with blunt thoracoabdominal trauma were included in the study. The mean age of the study population was 37.2 years, and the majority of patients were male (93.3%), reflecting the typical demographic profile of trauma patients. The baseline demographic characteristics and mechanisms of injury are summarized in Table 1.

**Table 1:** Baseline characteristics of study participants (n=120)

Variable	Value
Total number of patients	120
Mean age ± SD (years)	37.2 ± 11.4
Male	112 (93.3%)
Female	8 (6.7%)

The most common mechanism of injury was road traffic accidents, followed by falls and assault-related trauma. The mechanisms of injury in the study population are shown in Table 2.

**Table 2:** Mechanism of injury among study participants (n = 120) Values are presented as number (percentage).

Mechanism	Number of patients	Percentage
Road traffic accident	86	71.7%
Fall	25	20.8%
Assault	9	7.5%

Injury distribution

Based on the final hospital discharge diagnosis, thoracic injuries were identified in 78 patients (65%), while abdominal injuries were present in 48 patients (40%). Six patients sustained combined thoracoabdominal injuries. The distribution of injuries in the study population is shown in Table 3.

**Table 3:** Distribution of thoracic and abdominal injuries among study participants (n = 120) Six patients had combined thoracoabdominal injuries.

Injury type	Number of patients	Percentage
Thoracic injuries	78	65%
Abdominal injuries	48	40%

Detection of thoracic injuries

Extended focused assessment with sonography in trauma detected thoracic injuries in 68 of the 78 patients with confirmed thoracic trauma, while 10 patients with thoracic injuries had negative eFAST examinations, corresponding to a sensitivity of 87.2% (95% CI 77.9-93.4). A comparison between eFAST and chest radiography in detecting thoracic injuries is presented in Table 4.

**Table 4:** Detection of thoracic injuries by eFAST and chest radiography (n = 78) Comparison between eFAST and chest radiography detection rates was performed using McNemar’s test (p < 0.001). eFAST = extended focused assessment with sonography in trauma

Diagnostic modality	Injuries detected	Total thoracic injuries	Detection rate
eFAST	68	78	87.20%
Chest radiography	32	78	41.00%

Chest radiography detected thoracic injuries in 32 of the 78 patients, corresponding to a detection rate of 41.0%. In comparison, eFAST detected 68 thoracic injuries (87.2%). The difference in detection between eFAST and chest radiography was statistically significant (McNemar test, p < 0.001).

For abdominal trauma, eFAST detected injuries in 46 of the 48 patients with confirmed intra-abdominal pathology, while two patients with abdominal injuries had negative ultrasound examinations, corresponding to a sensitivity of 95.8% (95% CI 85.7-99.5). The diagnostic performance of eFAST for thoracic and abdominal injuries is summarized in Table 5.

**Table 5:** Diagnostic performance of eFAST for thoracic and abdominal injuries (n = 120) CI = confidence interval; PPV = positive predictive value; NPV = negative predictive value; eFAST = extended focused assessment with sonography in trauma

Parameter	Thoracic injury	Abdominal injury
Sensitivity	87.2% (95% CI 77.9–93.4)	95.8% (95% CI 85.7–99.5)
Specificity	100% (95% CI 91.6–100)	100% (95% CI 94.9–100)
Positive predictive value	100%	100%
Negative predictive value	80.8%	97.3%

Management outcomes

Based on clinical and imaging findings, intercostal chest tube insertion was performed in 61 patients, exploratory laparotomy was performed in 16 patients, and conservative management was undertaken in 43 patients.

**Figure 1:**
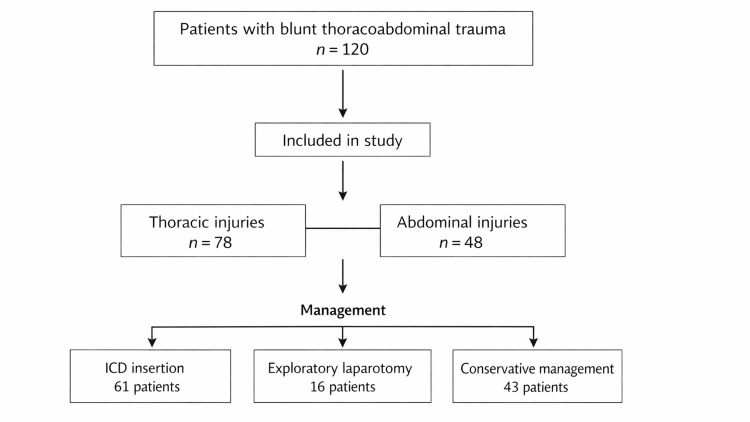
Flow diagram of patient inclusion and management outcomes Among 120 patients, 78 had thoracic injuries, and 48 had abdominal injuries; six patients had combined thoracoabdominal injuries. ICD = intercostal chest tube drainage

All patients who required intercostal chest tube insertion had positive thoracic ultrasound findings, while patients undergoing exploratory laparotomy had positive abdominal ultrasound findings, demonstrating a strong correlation between ultrasound findings and definitive management decisions. The overall patient inclusion, injury distribution, and management outcomes are illustrated in Figure 1.

## Discussion

The present prospective study evaluated the diagnostic utility of eFAST in hemodynamically stable patients presenting with blunt thoracoabdominal injury. The findings demonstrate that bedside ultrasound is a highly reliable diagnostic modality for the early detection of clinically significant thoracic and intra-abdominal injuries. In this study, eFAST demonstrated a sensitivity of 87.2% for thoracic injuries and 95.8% for abdominal injuries, with a specificity of 100% for both categories. These results highlight the value of ultrasound as a rapid bedside screening tool in trauma settings.

The sensitivity observed for thoracic injury detection in this study is comparable with findings from previous studies evaluating the extended FAST protocol. This similarity may be attributed to the use of standardized eFAST examination techniques and the ability of ultrasound to reliably detect pleural air and fluid. However, variations across studies may arise due to differences in patient selection, operator experience, and the use of computed tomography as a reference standard. In the present study, the inclusion of clinically suspected cases and reliance on a composite reference standard may have influenced the observed diagnostic performance. Kirkpatrick et al. first described the extension of the FAST examination to include thoracic assessment, demonstrating the ability of ultrasound to detect pneumothorax and hemothorax in trauma patients [4]. Subsequent studies have confirmed the high diagnostic performance of thoracic ultrasound in trauma settings and its superiority over conventional chest radiography in detecting pneumothorax [12,13]. In the present study, chest radiography identified only 41% of thoracic injuries, whereas eFAST detected 87.2%, supporting previous observations that supine chest radiographs may fail to detect small pneumothoraces and hemothoraces in trauma patients [11].

The diagnostic performance of ultrasound for intra-abdominal injury detection observed in this study is also consistent with existing literature. Earlier investigations evaluating point-of-care ultrasound in trauma settings have reported sensitivities ranging between 90% and 97% for the detection of clinically significant intra-abdominal fluid collections [6,7]. In our study, eFAST demonstrated a sensitivity of 95.8% for abdominal injuries, which falls within this range. These findings reinforce the established role of ultrasound in rapidly identifying hemoperitoneum and guiding early decision-making in trauma care. However, false-negative results may occur in cases of early or minimal intraperitoneal fluid, contained solid organ injuries, or hollow viscus injuries without significant free fluid. Additionally, ultrasound is inherently limited in detecting retroperitoneal injuries and may miss injuries that do not produce appreciable fluid collections. These limitations highlight the complementary role of computed tomography in stable patients for comprehensive injury evaluation.

Another important observation in this study was the strong correlation between ultrasound findings and subsequent management decisions. All patients requiring intercostal chest tube insertion had positive thoracic ultrasound findings, and patients undergoing exploratory laparotomy had positive abdominal ultrasound findings. This highlights the clinical relevance of eFAST in identifying injuries that require urgent intervention. Previous studies have similarly demonstrated that early ultrasound evaluation can significantly influence trauma management by facilitating timely operative or procedural intervention [8,14].

The findings of the present study are particularly relevant in resource-limited healthcare settings where access to advanced imaging modalities may be restricted. Computed tomography remains the gold standard for detailed injury characterization in trauma patients; however, it may not always be immediately available in high-volume emergency departments or resource-constrained environments [10]. Bedside ultrasound provides several advantages in such settings, including rapid availability, repeatability, and absence of ionizing radiation. However, in the absence of routine CT correlation, it is possible that certain injuries not associated with significant fluid or immediate clinical manifestations may have been missed. This underscores that while eFAST is highly effective for identifying clinically significant injuries requiring intervention, it may not detect the full spectrum of injuries, particularly those without free fluid or involving the retroperitoneum. As highlighted by previous investigations conducted in low- and middle-income countries, point-of-care ultrasound can significantly improve diagnostic accuracy and triage efficiency in trauma care [10,15].

Despite the high diagnostic accuracy observed in this study, several limitations should be acknowledged. First, this study was conducted at a single tertiary care center and involved a relatively limited sample size. Second, computed tomography was not routinely performed in all patients; instead, the final diagnosis was based on a composite reference standard including operative findings, clinical progression, and follow-up imaging when indicated. While this reflects real-world trauma management practices, it may introduce verification bias. Finally, ultrasound is an operator-dependent modality, and diagnostic accuracy may vary depending on the experience and training of the examiner.

Overall, the findings of this study support the growing body of evidence indicating that eFAST is an effective and reliable diagnostic modality for the rapid evaluation of blunt thoracoabdominal trauma. By facilitating early detection of clinically significant injuries and guiding management decisions, bedside ultrasound plays a crucial role in modern trauma care.

## Conclusions

Extended focused assessment with sonography in trauma (eFAST) is a valuable bedside tool for the evaluation of hemodynamically stable patients with blunt thoracoabdominal trauma. In this study, eFAST demonstrated high sensitivity for detecting clinically significant thoracic and abdominal injuries and significantly outperformed chest radiography in identifying thoracic trauma. The observed association between ultrasound findings and subsequent management decisions highlights its utility as a rapid triage adjunct in trauma care.

Given its immediate availability, noninvasive nature, and lack of ionizing radiation, bedside ultrasound is particularly useful in high-volume emergency departments and resource-limited settings. However, in the absence of routine computed tomography correlation, eFAST may not detect the full spectrum of injuries, particularly those without associated free fluid or involving the retroperitoneum. Therefore, while computed tomography remains the gold standard for comprehensive evaluation, eFAST serves as an effective initial screening modality when used in conjunction with clinical assessment.
